# SPD_0410 negatively regulates capsule polysaccharide synthesis and virulence in *Streptococcus pneumoniae* D39

**DOI:** 10.3389/fmicb.2024.1513884

**Published:** 2025-01-03

**Authors:** Ye Tao, Li Lei, Shuhui Wang, Xuemei Zhang, Yibing Yin, Yuqiang Zheng

**Affiliations:** ^1^Department of Clinical Laboratory, Children’s Hospital of Chongqing Medical University, National Clinical Research Center for Child Health and Disorders, Ministry of Education Key Laboratory of Child Development and Disorders and Chongqing Key Laboratory of Pediatric Metabolism and Inflammatory Diseases, Chongqing, China; ^2^Dujiangyan People’s Hospital, Chengdu, China; ^3^Key Laboratory of Diagnostic Medicine Designated by the Ministry of Education, Chongqing Medical University, Chongqing, China

**Keywords:** *Streptococcus pneumoniae*, capsular polysaccharides, transcriptional regulation, carbohydrate metabolism, virulence

## Abstract

*Streptococcus pneumoniae* capsular polysaccharide (CPS) is a crucial virulence factor for this pathogenic bacterium and is partially under transcriptional control. In this study, we used electrophoretic mobility shift assays and DNA enzyme footprinting to identified the hypothetical protein SPD_0410 as a negative regulator of *cps* locus. Our results showed that the D39Δ*spd0410* mutant strain exhibited significantly elevated CPS levels compared to the parental strain D39s. SPD_0410 directly binds at two specific sites on the *cps* promoter. The regulatory effect of SPD_0410 on CPS was weakened after the mutation of specific binding sites in the promoter. RNAseq analysis revealed that the deletion of *spd0410* led to alterations in glucose metabolism. However, the altered glucose levels appeared to eliminate the regulation of CPS synthesis by SPD_0410. Deleting the *spd0410* gene resulted in higher invasion and phagocytic resistance of bacteria and *in vivo* mouse experiments confirmed that D39Δ*spd0410* caused more severe systemic disease than the parental strain D39s. Our results indicated that SPD_0410 negatively regulates the synthesis of *S. pneumoniae* capsules and can directly alter pneumococcal virulence.

## 1 Introduction

*Streptococcus pneumoniae* is a common opportunistic pathogen that asymptomatically colonizes the human upper respiratory tract. However, this process may result in severe invasive diseases including pneumonia, meningitis, and bacteremia in immunocompromised or environmentally stressed patients (Bogaert et al., 2004). *S. pneumoniae* pathogenicity stems from its ability to evade immune system clearance and adapt within the host thus contributing to its status as a major global public health concern (O’Brien et al., 2009; Prina et al., 2015; Andre et al., 2017). This pathogenicity of *S. pneumoniae* is also contingent upon colonization and virulence factors including capsular polysaccharides (CPS), surface proteins, and proteases (Kadioglu et al., 2008; Mitchell and Mitchell, 2010). In particular, the CPS is a pivotal virulence determinant due to its dual role in protection from host immune defenses and in promoting efficient colonization and invasion of host tissues (Kadioglu et al., 2008; Geno et al., 2015). Regulation of CPS synthesis includes transcriptional regulation of *cps* promoters (Shainheit et al., 2014; Wen et al., 2015; Wen et al., 2016), epigenetic regulation of phase variation (Wang et al., 2020; Zhang et al., 2021), and post-translational modification of the bacterial tyrosine kinase system CpsBCD (Morona et al., 2000; Nourikyan et al., 2015; Nakamoto et al., 2021).

Transcriptional regulation of capsular expression plays a crucial role in the pathogenesis of pneumococcal disease and is a key pathway that modulates CPS production (Shainheit et al., 2014; Wen et al., 2015; Wu et al., 2016). The core −10 and −35 motifs and the spacing between these elements are key regulators of the transcriptional response (Shainheit et al., 2014; Wen et al., 2015; Wen et al., 2016). The involvement of these promoter elements has been illustrated for the serotype 2 strain D39 (Wen et al., 2015) and the serotype 4 strain TIGR4 (Shainheit et al., 2014). Additionally, the absence of the core promoter elements results in a marked reduction in CPS production and a concomitant decline in virulence in murine experimental infection models (Wen et al., 2015).

Regulatory factors that directly interact at the *cps* promoter sequence are only partially defined (Moscoso and García, 2009) but include ComE (Zheng et al., 2017), ComX (Luo and Morrison, 2003), MgaSpn (Xiao et al., 2021), RegM (Giammarinaro and Paton, 2002), and CpsR (Wu et al., 2016). These regulators perform differing functions but all are necessary for the full extent of capsule synthesis. For instance, the phosphorylated form of ComE negatively regulates *cps* gene cluster expression *in vivo* (Zheng et al., 2017). The MgaSpn protein in *S. pneumoniae* is orthologous to the Mga/AtxA family member Mga found in *Streptococcus pyogenes* and functions as an environmental sensor to regulate capsule and teichoic acid production (Xiao et al., 2021). In addition, *cps* transcriptional regulators from serotype 3 *S. pneumoniae* strains can function in non-serotype 3 strains indicating a shared regulatory mechanism across strains (Moscoso and García, 2009).

Our laboratory had previously identified a novel CPS regulatory factor SPD_0410 in serotype 2 strain D39 (Wu et al., 2016). In the current study, we further characterized this protein and found it functioned as a negative regulator of CPS production. This protein directly bound the *cps* promoter to modulate CPS expression and influenced bacterial virulence. We also linked SPD_0410 to regulation of glycolysis and gluconeogenesis and the presence of glucose eliminated the regulatory ability of SPD_0410. This study links SPD_0410 with the CPS regulatory network and highlights its role in virulence and provides novel perspectives and insights into the exploration of hypothetical proteins associated with *S. pneumoniae* virulence.

## 2 Materials and methods

### 2.1 Bacterial strains and growth conditions

The bacterial strains and plasmids used in this study are listed in Table 1. *S. pneumoniae* strain D39 derivatives were cultured in C+Y medium (C medium supplemented with 5% yeast extract pH 7.0) in the presence or absence of glucose as sole carbon source as required or on blood agar plates at 37°C in a 5% CO2 atmosphere. Antibiotics were used at the following concentrations: erythromycin 100 μg/mL. streptomycin 150 μg/mL, kanamycin 200 μg/mL as well as spectinomycin 50 μg/mL for *Escherichia coli* and 200 μg/mL for *S. pneumoniae*.

**TABLE 1:** Strains and plasmids.

Strain/plasmid	Relevant genotype/phenotype	Antibiotic	Reference/source
**Strain**			
D39	*S. pneumoniae* strain D39, serotype 2, encapsulated		NCTC
D39s	D39 derivative; *rpsL1*	Sm^R^	Lab stock
D39Δ*spd0410*	D39 derivative; D39Δ*spd0410*	Erm^R^	This study
D39Δ*spd0410*Com	D39Δ*spd0410*::pPEPZ-*spd0410*	Spec^R^	This study
D39s-P*cps*-luc	D39s::pPTH3937, D39s with Luc under the control of *cps* promoter	Chl^R^	This study
D39Δ*spd0410*-P*cps*-luc	D39Δ*spd0410*::pPTH3937, D39Δ*spd0410* with Luc under the control of *cps* promoter	Chl^R^	this study
D39Δ*spd0410*Com-P*cps*-luc	D39Δ*spd0410*Com::pPTH3937, D39Δ*spd0410*Com with Luc under the control of *cps* promoter	Chl^R^	This study
D39ΔP*cps*::Jc	D39s, ΔP*cps*::JC, the *cps* region is replaced with Janus cassette	Kana^R^	This study
D39s-P*cps*-Mut	D39s with the P*cps* multi sites mutation	Sm^R^	This study
D39s-P*cps*-Mut 1	D39s with the P*cps* mutation 1	Sm^R^	This study
D39s-P*cps*-Mut 2	D39s with the P*cps* mutation 2	Sm^R^	This study
**Plasmid**			
pPEPZ-Plac	Cloning vector	Spec^R^	Lab stock
pPEPZ-*spd0410*	Plasmid contain *spd0410*	Spec^R^	This study
pTH3937	P*cps* luciferase reporter plasmid	Chl^R^	Lab stock
pPAL7	Protein expression vector	Amp^R^	Lab stock
pPAL7-*spd0410*	Plasmid expressing SPD_0410 protein	Amp^R^	This study

### 2.2 Construction of mutants

Mutants were generated via homologous recombination using standard techniques using the primers specified in Supplementary Table 1. All strains employed in this study were derived from D39s, a streptomycin-resistant *S. pneumoniae* serotype 2 variant (Zhang et al., 2021). Construction of mutant strain D39Δ*spd0410* utilized D39s genomic DNA as a template. In brief, primers P1/P2 and P3/P4 were used to amplify the upstream and downstream fragments of the *spd0410* gene by PCR and Erm F/Erm R primers to amplify the *erm* fragment with homologous arms at both ends. These three amplicons were ligated to generate a recombinant fragment that was then introduced into D39s by transformation. Individual colonies from blood agar plates containing erythromycin were isolated and mutants were screened via PCR using primers P1/P4 to identify erythromycin-resistant D39Δ*spd0410* mutants.

The plasmid pPEPZ and the entire *spd0410* gene fragment from above were double digested with *BgI* II and *Xho*I and ligated to produce pPEPZ–*spd0410*. This plasmid construct was then transformed into D39Δ*spd0410* to obtain the ectopic complemented strain D39Δ*spd0410*Com. The *spd0410* gene was integrated into the bacterial genome via plasmid pPEPZ, ensuring its proper expression and functionality within the genome.

To construct *cps* promoter (P*cps*) point mutants, we amplified the Janus cassette fragment (Sung et al., 2001) with primers Pr1332/Pr1333 as well as upstream and downstream regions of *cps* promoter fragments with primers Pr9020/9026 and Pr9027/Pr9023. These three amplicons were ligated and transformed into D39s to generate D39ΔP*cps*::JC. Subsequently, we used D39s as a template to amplify the upstream and downstream regions of *cps* promoter fragments with Pr9028/Pr9021 and Pr9022/Pr9029. We connected the two fragments to generate a P*cps* fragment of 7-point mutants. The fragment was then transferred to D39ΔP*cps*::JC to produce the D39P*cps*-mut 1 strain. As mentioned above, primers Pr9028/Pr9025 and Pr9024/Pr9029 were used to amplify the upstream and downstream fragments of *cps* promoter fragments containing 8-point mutations, and then the fragment was connected and transferred into D39ΔP*cps*::JC to produce D39P*cps*-mut 2 strain. D39P*cps*-mut 1 and D39P*cps*-mut 2 were used as templates to amplify upstream and downstream regions of the *cps* promoter using the primers Pr9028/Pr9034 and Pr9033/Pr9029, respectively. The two fragments were then ligated and transferred into the D39ΔP*cps*::JC strain resulting in the generation of the binding site double mutant strain D39P*cps* mut.

### 2.3 Evolutionary analysis

The protein BLAST tool used the amino acid sequence of SPD_0410 from *S. pneumoniae* D39 to search the NCBI database. Multiple sequence alignment and cluster analysis of the top-scoring proteins were conducted using Clustal-X 2.1. The evolutionary tree of the proteins was constructed using MEGA 11. Phylogenetic analysis was performed using the Neighbor-Joining method, which applied the Poisson model to calculate pairwise distances. Bootstrap support values (%) were calculated with 1000 replicates to assess the reliability of the tree.

### 2.4 Western blotting

Protein production was quantified from *S. pneumoniae* strains cultured in C+Y medium at 37°C / 5% CO_2_. The strains were harvested at logarithmic growth (OD_600nm_ = 0.5) and lysed using a 0.5% deoxycholate buffer. The samples were separated using Tris-glycine SDS-PAGE and wet transferred to PVDF membranes. The membrane was incubated with 5% skim milk powder at 37°C for 2 h, then washed three times with TBST (Tris-buffered saline with 1% Tween 20) for 15 min each. After washing, the membrane was incubated overnight at 4°C with the primary antibody targeting CPS type 2 (1:500) (Danish National Serum Institute, Copenhagen). The CPS type 2 antibody was a non-adsorbed polyclonal antibody against type 2 polysaccharides, and the excess non-specific components in cell wall components were removed by the method described earlier (Kant et al., 2023). Following the overnight incubation, the membrane was washed again three times with TBST and then incubated with the secondary antibody, either goat anti-rabbit IgG or goat anti-mouse IgA (both at 1:10,000 dilutions), at 37°C for 1 h. After washing the membrane again, the gel image was performed. Protein bands were visualized using Image Lab software (Bio-Rad, Hercules, CA, USA) after incubation with Immobilon Western horseradish peroxidase (HRP) substrate peroxide solution (Millipore, Burlington, MA, USA).

### 2.5 Enzyme-linked immunosorbent assay (ELISA)

Bacterial cells were collected during the logarithmic growth phase (OD_600nm_ = 0.5) and suspended in a sterile PBS. These residues were then inactivated in a 58°C water bath for 45 min to prepare cell wall samples. After gradient dilution, the samples were coated onto 96-well plates and incubated overnight at 4°C. The primary antibody used was CPS polyclonal antibody type 2 (1:500) (see above) followed by incubation with goat anti-rabbit IgG-HRP antibody (1:500) (KPL, Gaithersburg, MD, USA). Absorbance at 450 nm was measured after color development to quantify CPS content on the bacterial surface. The results of the representative experiment were presented as mean ± standard deviation of three replicates.

### 2.6 Determination of capsular glucuronic acid

Logarithmic growth phase samples were collected and washed twice with sterile PBS followed by an additional wash with Tris-MgSO_4_ to prepare the whole bacterial sample. Cell wall samples were prepared from bacterial precipitates, washed with sterile PBS, and then suspended in PBS containing pre-boiled 2% SDS and boiled at 100°C for 30 min. After cooling to room temperature, the suspension was centrifuged and the cell walls were washed 3 × with PBS to completely remove SDS. The resulting precipitate was suspended in 400 μL Tris-MgSO_4_ to which 40 μL TE buffer containing 30 mg/mL lysozyme was added for cell wall lysis. The quantification of glucuronic acid in cell walls or whole bacterial capsules was performed as previously described for the quantitative determination of uronic acid (Blumenkrantz and Asboe-Hansen, 1973).

### 2.7 Transmission electron microscopy (TEM)

Bacterial precipitates harvested during logarithmic growth in C+Y medium were washed twice with sterile PBS. Then the residues were gently resuspended in 1 mL 2.5% glutaraldehyde fixing solution added slowly along the tube wall to ensure minimal disruption to the bacteria and then fixed overnight at 4°C. Electron microscopy was performed by Xavier Biotechnology (Wuhan, China). Capsule thickness was determined by measuring 15 randomly chosen cells using Image J software.

### 2.8 Expression and purification of SPD_0410 protein

The *S. pneumoniae spd0410* gene was amplified by PCR and cloned into expression vector pPAL7 using *Eco*RI and *Xho*I double digests of amplicons (see above). The construct was transferred into *E. coli* BL21(DE3) for fusion protein expression. SPD_0410 recombinant protein was induced in a Luria-Bertani medium supplemented with 100 μg/mL ampicillin and 0.5 mM isopropyl-b-d-thiogalactoside (IPTG). The protein was purified using the commercial Profinity eXact system (Biorad, Hercules, CA, USA).

### 2.9 Electrophoretic mobility shift assay (EMSA)

Primers P*cps*F/P*cps*R were employed to amplify fragments of the *cps* promoter region from strain D39s (Supplementary Table 1). The binding experiment was initiated by incubating a 10 μL reaction mixture at 25°C for 10 min comprised of 1 μL binding buffer (10 mM Tris-HCl, pH 7.4, 1 mM dithiothreitol, 1 mM EDTA, 50 mM KCl, 5% glycerol, 50 mg/mL bovine serum albumin, 0.05% Nonidet P-40), 0.5 μg of poly(dI-dC), 0–6 μg of SPD_0410 protein, and 1 ng of labeled probe. Following this incubation, a 100-fold excess of unlabeled probes was added to serve as specific competitors in the cold probe reaction system. The mixture was then further incubated at 25°C for 20 min followed by electrophoresis through 5% native polyacrylamide gels in 1 × TBE buffer. Subsequent steps followed the protocol provided by the LightShift R Chemiluminescence EMSA Kit (Thermo Fisher, Pittsburg, PA USA).

### 2.10 DNase I footprinting

DNase I footprinting assays were performed as described previously (Wang et al., 2012). The resulting probe (300 ng) was briefly incubated with varying amounts of protein in a 40 μL reaction system at 25°C for 30 min. Following incubation, the reaction samples underwent enzyme digestion using 0.015 units of DNase I (Promega. Madison, WI, USA) at 25°C for 1 min. After enzyme digestion, the proteins were removed via phenol extraction, and the DNA was precipitated with ethanol. The precipitated DNA was dissolved in Milli-Q ultra-pure water and analyzed using an ABI sequencer and ABI GeneScan 500 Liz was used as the ladder.

### 2.11 RNA extraction and qRT-PCR

Total RNA was extracted from *S. pneumoniae* cultures harvested during the logarithmic (OD_600nm_ = 0.5) or the early growth (OD_600nm_ = 0.1) phases. Bacterial precipitates were suspended with 100 μL TE buffer containing 30 mg/mL lysozyme and incubated at 25°C for 25 min. Bacterial total RNA was subsequently extracted using a total RNA extraction kit designed for cultured cells/bacteria (Tiangen, Beijing, China). RNA (1 μg) was reverse transcribed into cDNA using the PrimeScript First strand cDNA synthesis kit (Takara Bio, Shiga, Japan). Real-time PCR was conducted on a CFX Connect system (Biorad) using the *gyrB* gene as the internal reference standard. All the primers used in the experiment are shown in Supplementary Table 1. The results of representative experiments were expressed as the mean ± standard deviation of three replicates.

### 2.12 Construction of luciferase reporter strains and luciferase assay

The integrating plasmid pTH3937 (Wen et al., 2015) containing the complete promoter region and firefly luciferase gene was transferred into D39s, D39Δs*pd0410*, and D39Δ*spd0410*Com strains using bacteria grown to OD_600__*nm*_ = 0.1 in C+Y medium at 37°C. The plasmid was supplied by Professor Jingren Zhang from Tsinghua University, Beijing. The fluorescence intensity (RLU) was measured by mixing 100 μL of bacterial culture with 0.66 μM fluorescein sodium salt. The results were normalized to the optical density (OD_600nm_) of the samples.

### 2.13 RNAseq analysis

*S. pneumoniae* strains D39s and D39Δ*spd0410* strains were cultured in C+Y medium to OD_600nm_ = 0.5. Bacterial total RNA was extracted using the RNAprep pure cell/bacterial kit (Tiangen, Beijing, China) followed by transcriptome sequencing conducted at Novogene Bioinformatics Technology (Beijing, China). The reference genome used was the *S. pneumoniae* D39s genome (GenBank Acc. No. NC_008533.2). Differential expression analysis of two groups (each with 3 biological replicates) was performed using the DESeq2 R package (version 1.20.0). Genes with adjusted *P*_*adj*_ < 0.05 identified by DESeq were classified as differentially expressed.

### 2.14 Adhesion and antiphagocytic assay

Adhesion and invasion assays of *S. pneumoniae* were conducted using the human type II lung epithelial cell line A549. Cells were seeded at a density of 2 × 10^5^ per well in 24-well plates and cultured overnight at 37°C. The confluent epithelial cell monolayers (2 × 10^5^ cells/well) were then inoculated with 2 × 10^7^ colony-forming units (CFU) of pneumococci at a multiplicity of infection (MOI) of 1:100. The cultures were incubated in Dulbecco’s Modified Eagle’s medium (DMEM) at 37°C with 5% CO_2_ for 1 h. After incubation, the cells were washed 5 × with sterile PBS and lysed by the addition of double-distilled water for the adhesion experiment. The resulting lysate was diluted and plated onto agar plates to enumerate bacteria inside and outside the cells. Invading bacteria were enumerated by exposing extracellular bacteria to 100 μg/mL penicillin and 10 g/mL gentamicin for 15 min, respectively. The number of intracellular bacteria was then determined using the abovementioned method.

Primary peritoneal macrophages from male C57BL/6 mice were extracted and isolated according to the method previously described (Zhong et al., 2019). After 4–5 days of paraffin injection into the peritoneal cavity of mice, macrophages were extracted from the peritoneal lavage and inoculated into 24-well plates at a density of 2 × 10^5^ cells/well. Phagocytosis was evaluated using primary peritoneal macrophages from a mic, employing a protocol identical to that of the adhesion experiment. The anti-phagocytosis rate was calculated using the following formulas:


Anti-phagocytosisrate(%)

$$\;=\frac{e\,x\,t\,r\,a\,c\,e\,l\,l\,u\,l\,a\,r\,C\,F\,U}{i\,n\,t\,r\,a\,c\,e\,l\,l\,u\,l\,a\,r\,C\,F\,U+e\,x\,t\,r\,a\,c\,e\,l\,l\,u\,l\,a\,r\,C\,F\,U}$$

### 2.15 Animal experiments

Male C57BL/6 mice (6–8 weeks old, weight 18–20 g) were procured from the Laboratory Animal Center of Chongqing Medical University. All animal experiments described in this study were approved by the Animal Care and Use Committee of Chongqing Medical University and were conducted strictly with the provisions of the Guidelines for the Care and Use of Experimental Animals.

To investigate the effects of SPD_0410 on the colonization of the mouse nasopharynx, mice were anesthetized by peritoneal injection of 2% pentobarbital sodium (50 mg/kg), and the bacteria were inoculated through the nasal cavity to establish a pneumonia model. Mice were randomly divided into two groups (*n* = 6) and each mouse was inoculated nasally with 2 × 10^7^ CFU/30 μL bacteria. Nasal lavage fluid, heart blood, spleen, and lung tissues were collected 48 h after infection. After proper dilution, the samples were placed on blood agar plates to determine CFU.

The role of SPD_0410 in systemic infection was explored by dividing the mice into two groups (*n* = 12). Each mouse was intranasally infected with 1 × 10^8^ CFU/30 μL of bacteria. Survival was monitored daily for 14 days. After the 14-day survival observation period, the surviving mice were anesthetized by intraperitoneal injection of 2% pentobarbital sodium (50 mg/kg) and euthanized via cervical dislocation.

Lung biopsy samples were collected from mice infected with sterile PBS, D39s, and D39Δ*spd0410* strains, sterile PBS simulated infection as a control. Lung tissues were initially fixed in 4% paraformaldehyde at 4°C for 48 h followed by embedding in paraffin and sectioning into 5 mm thick slices. Sections were subsequently stained with hematoxylin and eosin (H&E) using standard protocols.

### 2.16 Statistical analysis

All statistical analyses in this study were conducted using Prism 8 software (GraphPad Software, San Diego, CA, USA). The data were evaluated using either an unpaired two-tailed Student’s *t*-test or a non-parametric Mann–Whitney U test. Survival data were analyzed using the log-rank (Mantel-Cox) test. Statistical significance was defined as ***P < 0.001; **P < 0.01; *P < 0.05; ns, not significant.

## 3 Results

### 3.1 Multi amino acid sequence alignment and evolutionary tree analysis

**FIGURE 1:**
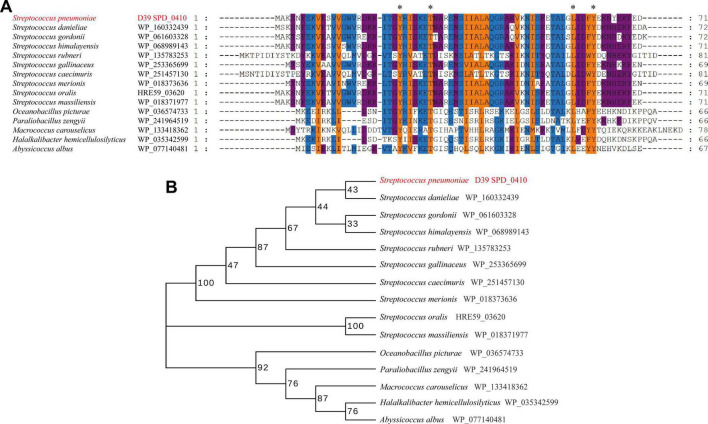
Conserved and evolutionary analysis of SPD_0410. **(A)** Multiple sequence alignment of SPD_0410 with homologous proteins in various bacterial species. The residues labeled with * represent the invariant residues. **(B)** Evolutionary tree of SPD_0410 protein and its homologous proteins.

SPD_0410 was previously identified as a potential transcriptional regulator that influenced CPS regulation in *S. pneumoniae* strain D39 (Wu et al., 2016). The protein is highly conserved among Gram-positive bacteria, particularly within the *Streptococcus* genus (Figure 1). However, these orthologs were primarily classified as hypothetical and their specific biological functions have yet to be thoroughly characterized. The data also revealed that the orthologous proteins SPD_0410, WP_035342599, and WP_077140481 possessed helix-turn-helix DNA binding domains (Carranza et al., 2024) suggesting that SPD_0410 may also have a function related to DNA binding.

### 3.2 SPD_0410 affects CPS production by down-regulation of the cps operon transcription

**FIGURE 2:**
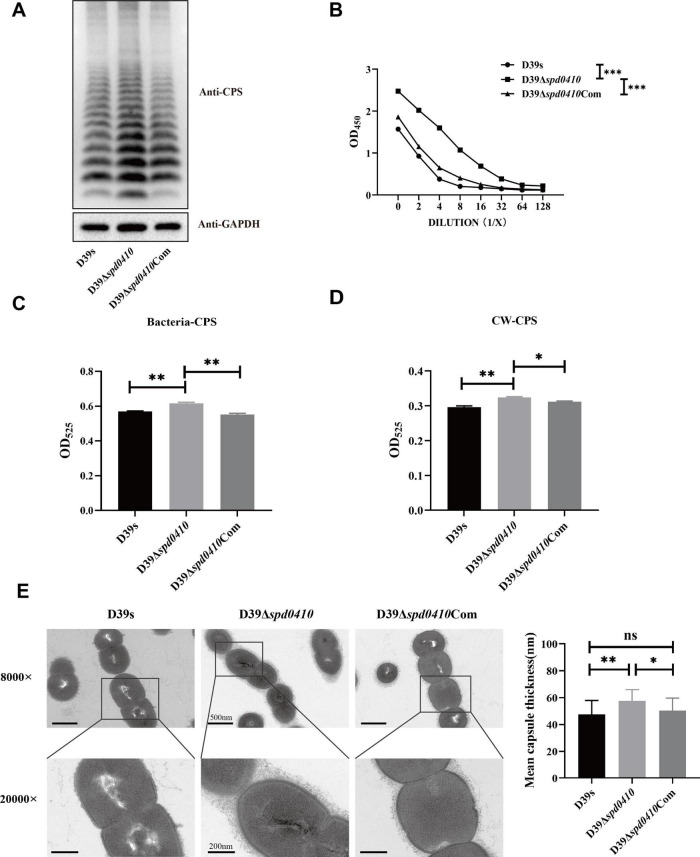
Effect of deletion of *spd0410* gene on CPS synthesis. **(A)** Western blotting detected CPS expression in the indicated strains’ whole-cell lysates. GADPH was used as a sample loading control. **(B)** ELISA determined the content of the capsule in bacterial lysate. Western blots and ELISA were probed with a rabbit anti-serotype 2 CPS polyclonal antibody. **(C,D)** Determination of capsule content by uronic acid assay. **(C)** Comparisons of whole-cell CPS levels. **(D)** Comparisons of cell wall-associated CPS levels. **(E)** Transmission electron microscopy of strains. The mean capsule layer diameters are indicated; *n* = 15. The average capsule thickness of D39s, D39Δ*spd0410* and D39Δ*spd0410*Com is 47.5 ± 10.4, 57.6 ± 8.4 and 50.3 ± 9.4 nm, respectively; The results of representative experiments are presented as the mean of three replicates ± SD. ***P < 0.001; **P < 0.01; *P < 0.05; ns, not significant, as analyzed by unpaired two-tailed Student’s *t*-test.

To investigate the role of SPD_0410 in CPS expression, we constructed the *spd0410* defective (D39Δ*spd0410*) and complemented (D39Δ*spd0410*Com) strains (Supplementary Figures 1A, B). The growth rates in C+Y medium for these strains did not significantly differ (Supplementary Figure 1C). We subsequently assessed the relative CPS levels in these strains and CPS levels in strain D39Δ*spd0410* were significantly elevated compared to the WT strain D39s. WT levels were also restored in the complemented strain D39Δ*spd0410*Com (Figure 2A). These differences were further verified using ELISA assays that quantified surface CPS levels (Figure 2B). The *S. pneumoniae* serotype 2 capsule comprises uronic acid (Kolkman et al., 1998) so we also quantified glucuronic acid levels in these strains. The mutant strain D39Δ*spd0410* produced whole cell and cell wall-associated uric acid levels significantly greater than the WT or complemented strains (Figures 2C, D). We also measured capsule thickness in these test strains using 15 capsules per group measured randomly. We found a significant increase in capsule thickness in the D39Δ*spd0410* strain (57.6 ± 8.4 nm) versus D39s (47.5 ± 10.4 nm) and D39Δ*spd0410*Com (50.3 ± 9.4 nm) (Figure 2E). These findings strongly indicated that deletion of the *spd0410* gene markedly enhanced CPS production and its presence at the bacterial cell surface.

**FIGURE 3:**
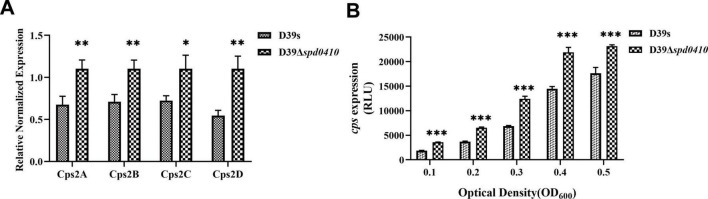
Transcriptional levels of *cps* gene clusters in D39s and D39Δ*spd0410* strains. **(A)** Relative mRNA levels of *cps*2A-D in early stages of bacterial logarithmic growth (OD_600nm_ = 0.1), the first four genes downstream of the *cps* operon in the strains. Relative mRNA levels are expressed relative to that of *gyrB*. **(B)** Luciferase activity analysis evaluated the expression of *cps* promoters in D39s and D39Δ*spd0410* strains. The results of representative experiments are presented as the mean of three replicates ± SD. ***P < 0.001; **P < 0.01; *P < 0.05; ns, not significant, as analyzed by unpaired two-tailed Student’s *t*-test.

We further examined the regulation of the *cps* operon by monitoring steady-state mRNA expression of the *cps*2A-D genes to ascertain whether SPD_0410 regulates the expression of the *cps* operon. We found no significant differences in *cps* operon expression between D39s and D39Δ*spd0410* during logarithmic growth (OD_600nm_ = 0.5) (Supplementary Figure 2). In contrast, the D39Δ*spd0410* strain displayed higher levels of mRNA for *cps*2A-D genes versus D39s at early growth stages (OD_600nm_ = 0.1) (Figure 3A). Together, these results indicated that SPD_0410 is a negative regulator that is may also linked to metabolic and resource allocation mechanisms of bacterial growth. Deletion of *spd0410* could probably activate specific transcriptional regulatory mechanisms in the early growth phase to facilitate environmental adaptation, altering the *cps* gene cluster levels and making new adjustments during logarithmic growth.

To characterize the activity of the *cps* promoter region during growth, the firefly luciferase gene was fused with the *cps* promoter and introduced into the D39s and D39Δ*spd0410* strains. We found that *cps* promoter activity was significantly enhanced in D39Δ*spd0410* (Figure 3B). This result was not entirely consistent with the mRNA data likely because the luciferase reporter assay only reflected the activity of the *cps* promoter region and did not fully capture the actual decay or stabilization mechanisms of the mRNA itself. However, it was clear that SPD_0410 negatively regulates CPS biosynthesis.

### 3.3 SPD_0410 specifically binds to the cps promoter region and regulates CPS production

**FIGURE 4:**
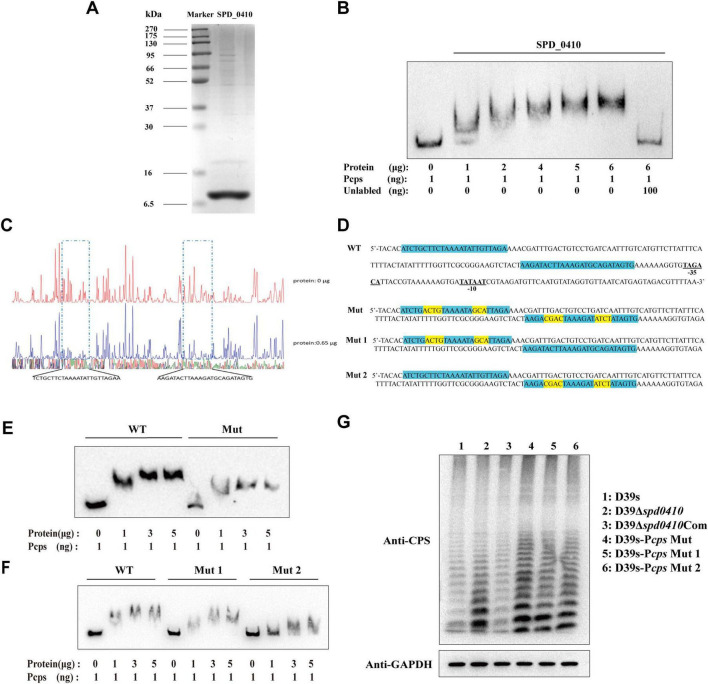
Electrophoretic mobility shift assay (EMSA) determines SPD_0410 binding to the P*cps*. **(A)** SDS-PAGE analysis of the SPD_0410 protein. **(B)** EMSA of P*cps* using the SPD_0410 protein, and 100 ng of non-biotin-labeled was added to compete with the labeled probe. **(C)** DNase I footprinting protection assay of SPD_0410. **(D)** Structural organization of the *cps* promoter-proximal region (WT). The binding sites for SPD_0410 are shown in blue. Mutated sequences are shown in yellow (Mut, Mut 1, and Mut 2). **(E)** Binding of SPD_0410 to the P*cps* and P*cps* Mut fragments. **(F)** Binding of SPD_0410 to the P*cps* Mut 1 and P*cps* Mut 2 fragments. **(G)** Western blotting was used to detect the expression of CPS in whole-cell lysates of the indicated strains.

To ascertain whether SPD_0410 specifically binds to the *cps* promoter region *in vitro*, we initially expressed and purified the SPD_0410 protein from *S. pneumoniae* using a heterologous expression system (Figure 4A). DNA electrophoretic mobility shift assays (EMSA) using 5′-biotin-labeled 218-bp long *cps* promoter probes indicated that electrophoretic mobility of the protein-DNA complexes decreased as the SPD_0410 protein concentration was increased (Figure 4B). Moreover, the introduction of unlabeled cold probes into the binding mixture led to reduced migration of the protein-DNA complexes (Figure 4B). These results indicated that SPD_0410 specifically binds to the *cps* promoter region. We additionally performed DNase I footprinting using the same 218-bp long *cps* promoter fragment labeled with FAM to determine the binding site more precisely (Figure 4C). We identified two protected sites on the *cps* promoter when SPD_0410 was present at 0.65 μg: a 24-bp region (5′-TCTGCTTCTAAAATATTGTTAGAA-3′) and a 25-bp region (5′-AAGATACTTAAAGATGCAGATAGTG-3′). These regions were upstream of the −35 and −10 boxes on the *cps* promoter (Figure 4D).

To further confirm that SPD_0410 regulates CPS expression through specific binding to the *cps* promoter, we generated gene constructs containing 7 or 8 mutations at two distinct binding sites *in vitro* in the *cps* promoter sequences: Mut (the motif CTTCTAAAATATTG was mutated to ACGTTAAAATAGCA and the motif TACTTAAAGATGCAG was mutated to CGACTAAAGATATCT), Mut 1 (the motif CTTCTAAAATATTG was mutated to ACGTTAAAATAGCA) and Mut 2 (the motif TACTTAAAGATGCAG was mutated to CGACTAAAGATATCT) (Figure 4D). The binding affinity of SPD_0410 for the mutant P*cps* probes was significantly reduced compared to the unmutated probe (Figures 4E, F). These data indicated that SPD_0410 specifically binds to the *cps* promoter.

Deletion or mutation of the −35 and −10 boxes within the core region of the *cps* promoter results in a substantial reduction in CPS production (Shainheit et al., 2014). We observed that SPD_0410 specifically binds to an area of the *cps* promoter located upstream of the −35 and −10 boxes (Figure 4D). We assumed the mutations we performed would not reduce CPS production. We introduced these point mutations into the bacterial genomes to test this hypothesis. We found that CPS was expressed at higher levels in D39s-P*cps*-Mut, D39s-P*cps*-Mut 1, and D39s-P*cps*-Mut 2 compared to D39s (Figure 4G). This finding further reinforces the notion that SPD_0410 directly binds to the *cps* promoter and negatively regulates its expression, thereby impacting CPS synthesis. Additionally, these results complement previous studies and highlight the critical role of the *cps* promoter core region in regulating CPS production (Kadioglu et al., 2008).

### 3.4 SPD_0410 affects the glucose metabolism pathway

**FIGURE 5:**
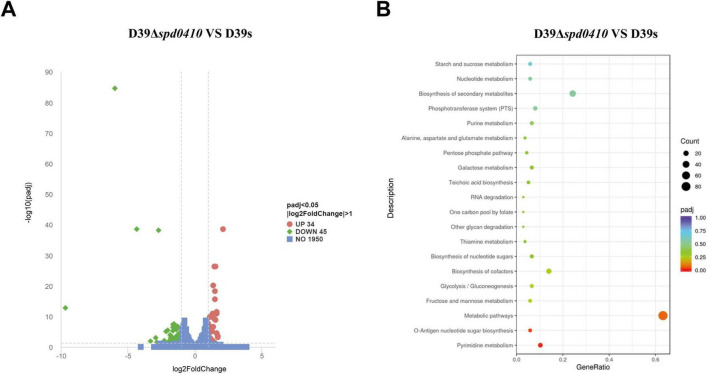
Comparison of differentially expressed genes between D39s and D39Δ*spd0410* by RNAseq analysis. **(A)** Volcano plot of differentially expressed genes. The abscissa represents the ratio of differentially expressed genes in D39Δ*spd0410* versus D39s; the ordinate represents the *P*-value between the two groups. **(B)** GO enrichment cluster analysis of differential genes.

To further elucidate the reasons behind the increased CPS levels resulting from the deletion of the *spd0410* gene and to investigate its complete functionality, transcriptome sequencing was performed on both D39s and D39Δ*spd0410* strains. We found 79 transcripts that were significantly differentially expressed based on a cutoff value of 1.0-fold change and a *P*_*adj*_ value of 0.05 in D39Δ*spd0410*. In particular, 34 genes were increased and 45 genes were decreased (Figure 5A). The up-regulated genes were primarily associated with pyrimidine metabolism and virulence factors whereas the down-regulated genes were linked to the ABC transport system, the phosphotransferase system (PTS), and glucose metabolism (Table 2). For instance, enzyme II genes of the PTS responsible for mediating the internalization of extracellular carbohydrates (Marciniak et al., 2012) were down-regulated by 1.2-fold to 2.0-fold in D39Δ*spd0410* (Table 2). Glucose is the preferred carbon source for *S. pneumoniae* and is transported intracellularly by the mannose-type PTS (*ma*n*LMN*) (Carvalho et al., 2011; Bidossi et al., 2012). Our data indicated that the *manLMN* genes such as *gadE*, *gadV*, and *gadW* were significantly down-regulated by 1.7-fold to 1.9-fold in the D39Δ*spd0410* mutant (Table 2). These genes are also under carbon catabolic repression via CcpA, which inhibits the uptake and metabolism of non-preferred sugars by binding to catabolic response elements (*cre*) (Görke and Stülke, 2008; Carvalho et al., 2011). However, the levels of *ccpA* did not differ between the D39s and D39Δ*spd0410* (data not shown), suggesting that SPD_0410 and CcpA may interact synergistically in regulating carbon catabolism.

**TABLE 2:** Differential gene expression for D39Δ*spd0410* relative to the D39s by RNAseq analysis.

Gene id	Gene	Description	log_2_ fold change	*P*-value
**Increased**				
**Pyrimidine metabolism**				
SPD_RS02910	spd_0543	Uracil-DNA glycosylase family protein	1.4918	1.39E-18
SPD_RS03280	pyrE	Orotate phosphoribosyltransferase	1.48850	2.13E-21
SPD_RS03275	pyrF	Orotidine-5′-phosphate decarboxylase	1.44978	1.43E-29
SPD_RS04580	pyrK	Dihydroorotate dehydrogenase electron transfer subunit	1.33072	8.98E-14
SPD_RS06050	pyrR	Bifunctional pyr operon transcriptional regulator/uracil phosphoribosyltransferase	1.03984	1.67E-08
**Virulence factor**				
SPD_RS08060	rgg1518	Rgg/GadR/MutR family transcriptional regulator Rgg1518	1.24744	3.18E-08
SPD_RS08455	mgaSpn	Virulence factor transcriptional regulator MgaSpn	1.10103	1.20E-12
SPD_RS09140	spd_1717	LytTR family DNA-binding domain-containing protein	1.01031	6.06E-06
SPD_RS10595	dltB	D-alanine–poly (phosphoribitol) ligase subunit DltB	1.00495	2.35E-10
SPD_RS10600	dltA	Teichoic acid D-Ala incorporation-associated protein DltA	1.09227	9.57E-05
**Decreased**				
**ABC transfer system**				
SPD_RS00475	spd_0089	Carbohydrate ABC transporter permease	−1.31952	0.001258
SPD_RS00485	spd_0090	ABC transporter substrate-binding	−2.16055	2.30E-07
SPD_RS05200	spd_0966	Sugar ABC transporter permease	−1.05367	2.61E-07
SPD_RS07935	satC	Carbohydrate ABC transporter	−1.25945	2.60E-06
SPD_RS07940	satB	Sugar ABC transporter permease	−1.20096	0.000206
**Phosphotransferase system**				
SPD_RS00335	gadV	PTS system mannose/fructose/N-acetylgalactosamine-transporter subunit IIB	−1.76673	9.74E-06
SPD_RS00340	gadW	PTSmannose/fructose/sorbose/N- PTS system mannose/fructose/sorbose family transporter subunit IID	−1.87046	3.00E-06
SPD_RS00345	gadE	PTS sugar transporter subunit IIA	−1.69409	3.80E-05
SPD_RS00350	gadF	PTS sugar transporter subunit IIA	−2.01639	9.26E-08
SPD_RS03020	spd_0560	PTS sugar transporter subunit IIB	−1.92547	0.001491
SPD_RS03025	spd_0561	PTS galactitol transporter subunit IIC	−1.23677	0.000476
**Glucose metabolism**				
SPD_RS00330	bgaC	beta-galactosidase	−1.16233	0.000450
SPD_RS00360	galM	Galactose mutarotase	−1.56759	0.000132
SPD_RS01445	adhP	Alcohol dehydrogenase AdhP	−1.04334	0.005366
SPD_RS02705	bglA-2	Glycoside hydrolase family 1 protein	−1.38018	4.89E-08
SPD_RS05615	lacG	6-phospho-beta-galactosidase	−1.05771	0.001894
SPD_RS10415	spd_1968	Beta-N-acetylhexosaminidase	−1.18103	0.003663
SPD_RS10430	spd_1971	Glycoside hydrolase family 125 protein	−1.15088	0.002722

*S. pneumoniae* derives most of its ATP from glycolysis (Bättig and Mühlemann, 2008) where accumulated NADH is re-oxidized by NADH oxidase using oxygen and this process enhances glycolytic efficiency. Our data revealed changes in the expression of several glycolytic- and gluconeogenesis-related enzymes including *galM*, *bglA-2*, *adhP*, and *spd1971* that were up-regulated by 1.0-fold to 1.6-fold (Figure 5B and Table 2). This indicated that intracellular energy metabolism is altered in D39Δ*spd0410* and links SPD_0410 to glucose transport and metabolism.

### 3.5 Glucose levels affect SPD_0410 regulation on CPS

Our transcriptome results indicated that SPD_0410 can alter glucose metabolism (Figure 5B), so we examined whether glucose levels affected the growth of D39s and D39Δ*spd0410*. We cultured strains in C+Y medium with glucose as the sole carbon source at levels of 4 mM, 8 mM, and 16 mM. We observed the growth of the strains at different glucose concentrations and found no significant differences between the two strains (Supplementary Figure 3). This indicated that D39Δ*spd0410* has strong adaptability, allowing it to rapidly sense changes in glucose levels in the environment and adjust cell metabolism accordingly. Consistent with these findings, bacterial morphologies were similar for both strains at all glucose levels, including in the absence of glucose (Supplementary Figure 4).

**FIGURE 6:**
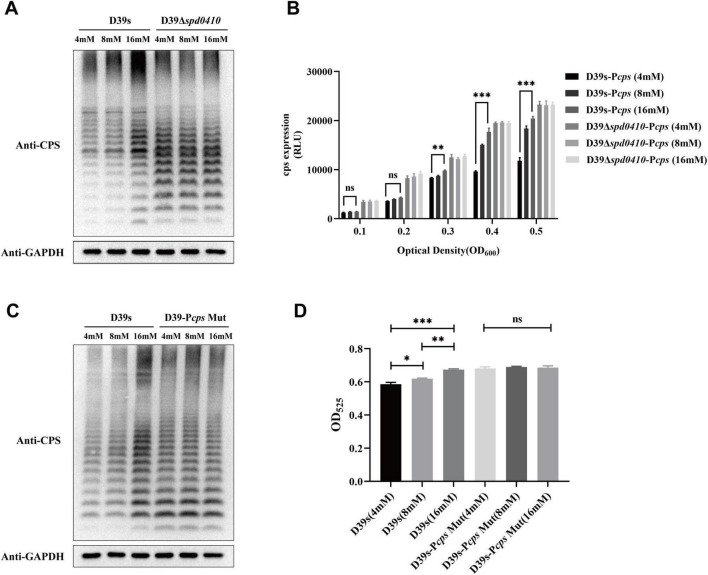
Glucose level affects CPS expression. **(A)** The expression of CPS in D39s and D39Δ*spd0410* strains at different glucose concentrations was detected by western blotting. **(B)** Luciferase activity analysis evaluated the expression of *cps* promoters in D39s-P*cps* and D39Δ*spd0410-*P*cps* strains at different glucose concentrations. The results of representative experiments are presented as the mean of three replicates ± SD. ***P < 0.001; **P < 0.01; ns, not significant, as analyzed by as analyzed by unpaired two-way ANOVA. **(C)** The expression of CPS in D39s and each mutant strain in different glucose concentrations was detected by western blotting. **(D)** Determination of whole-cell CPS levels in D39s and D39Δ*spd0410* strains at different glucose concentrations. The results of representative experiments are presented as the mean of three replicates ± SD.***P < 0.001; **P < 0.01; *P < 0.05; ns, not significant, as analyzed by unpaired two-tailed Student’s *t*-test.

We further examined whether glucose might influence the regulation of CPS by SPD_0410. CPS levels were elevated as glucose levels were increased for the D39s strain but not the D39Δ*spd0410* strain (Figure 6A). The activity of the *cps* promoter in D39s-P*cps* was increased as the glucose levels were increased. Still, this pattern was not observed in D39Δ*spd0410*-P*cps* (Figure 6B). In addition, we evaluated the CPS levels in the P*cps* multi-site mutation strain D39s-P*cps*-Mut and the results mirrored those of the mutant D39Δ*spd0410* (Figures 6C, D). These findings indicated that glucose is a regulator of SPD_0410. However, the specific mechanism needs to be further investigated.

### 3.6 Deletion of *spd0410* influences adhesion and pathogenicity of *S. pneumoniae*

**FIGURE 7:**
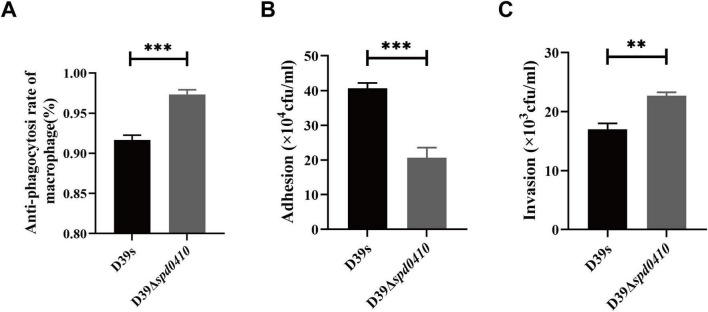
Macrophage phagocytosis and infection of epithelial cells. **(A)** Comparison of the ability between D39s and D39Δ*spd0410* against phagocytosis from mouse peritoneal primary macrophages. The cells were infected at an MOI of 100 with the indicated strains. **(B,C)** Infection of epithelial A549 cells. The cells were infected at an MOI of 100 with the indicated strains. **(B)** The number of bacterial adhesion to A549 cells. **(C)** The number of bacterial invasions to A549 cells. ***P < 0.001; **P < 0.01, analyzed by unpaired two-tailed Student’s *t*-test.

Due to the pivotal role of the capsule in the pathogenesis of *S. pneumoniae*, we subsequently investigated the impact of the SPD_0410 on bacterial virulence. The capsule effectively aids the bacteria in evading phagocytosis by the host immune system, so we examined our test strains in phagocytic evasion assays using primary mouse peritoneal macrophages. We found that D39Δ*spd0410* exhibited increased resistance to phagocytosis compared to D39s (Figure 7A). Additionally, adhesion to A549 cells was decreased in the mutant D39Δ*spd0410* (Figure 7B). In contrast, the D39Δ*spd0410* strain demonstrated enhanced invasion capabilities and penetrated the A549 cells monolayers more effectively than D39s (Figure 7C).

### 3.7 SPD_0410 is involved in systemic virulence and nasopharyngeal colonization of *S. pneumoniae*

**FIGURE 8:**
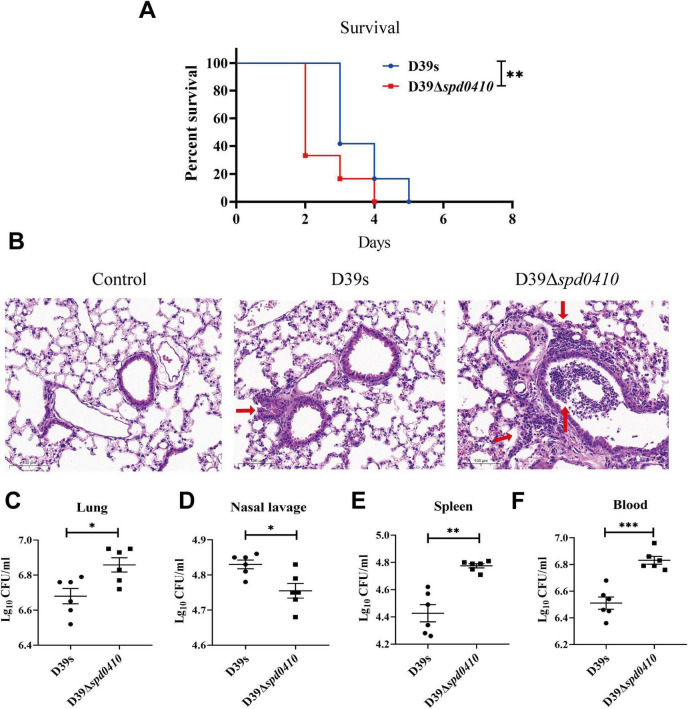
Survival of infected mice and bacterial colonization. **(A)** The survival curves of D39s and 39Δ*spd0410* strains infected mice via intranasal inoculation of 1 × 10^8^ CFU *S. pneumoniae* into C57BL/6 mice (*n* = 12 animals per group). Survival was analyzed using log-rank comparisons. **P < 0.05. **(B)** Photomicrograph of H&E-stained lung tissues of mice 48 h after bacterial infection in mice at the same dose as the colonization experiments. Red arrows indicate the inflammatory cells. **(C–F)** Intranasal infection with 2 × 10^7^ CFU of *S. pneumoniae* to observe bacterial colonization (*n* = 6 animals per group). Bacterial loads were evaluated by cultures of **(C)** lung homogenates, **(D)** nasal lavage, **(E)** spleen homogenates, and **(F)** blood. The results of representative experiments are presented as the mean of three replicates ± SD. ***P < 0.001; **P < 0.01; *P < 0.05, as analyzed by the non-parametric Mann–Whitney test.

To thoroughly investigate the impact of SPD_0410 on the infectivity of *S. pneumoniae*, we performed an *in vivo* assessment of infection in a mouse model using intranasal administration of the bacteria to establish a pneumonia model. We found that mice infected with D39Δ*spd0410* had a significantly reduced survival compared to the parental strain D39s (Figure 8A). We further examined the invasion capabilities of D39s and D39Δ*spd0410* strains in the mice, observed pathological lung damage, and measured bacterial loads after 48 h. H&E staining revealed greater inflammatory cell infiltration and more pronounced pathological damage in the D39Δ*spd0410* infection group (Figure 8B). We also observed increased bacterial colonization in the lungs of mice infected with D39Δ*spd0410* (Figure 8C). CFU in the nasopharyngeal lavage solutions of D39Δ*spd0410*-infected mice was lower than that of D39s-infected (Figure 8D), and this was consistent with the decreased adhesion levels for the mutant to lung epithelial cells (Figure 7B). In addition, mice infected with D39Δ*spd0410* showed greater susceptibility to invasion of the spleen and blood (Figures 8E, F).

Overall, these findings are consistent with the *in vitro* virulence experiments and indicate that SPD_0410 is a negative regulator of bacterial virulence that works by altering CPS levels on the bacterial cell surface.

## 4 Discussion

The *S. pneumoniae* capsule is crucial for bacterial virulence and has historically provided the first example of CPS linkage to virulence as well as to the transfer of genetic information via DNA (Griffith, 1928). CPS synthesis occurs through two primary pathways: single sugar polymerization (synthase-dependent) and discrete repeat unit assembly (Wzx/Wzy-dependent) (Yother, 2011). The synthase-dependent pathway progressively adds sugar through a single enzyme and occurs in serotypes 3 and 37 (Engholm et al., 2017). Conversely, other serotype strains follow the Wzx/Wzy-dependent pathway, synthesizing repeat units by adding sugars to the inner layer of the cell membrane and transferring sugar chains to the outer layer by the Wzy polymerase (Whitfield et al., 2020). In the Wzy synthesis pathway, the *cps* gene forms an operon between two genes not involved in capsule biosynthesis; *dexB*, and *aliA*. The first four genes in the CPS operon (*cps*ABCD), are widely conserved but their exact functions have not been fully elucidated (Kolkman et al., 1998; Yother, 2011).

Previous studies have reported that the *cpsA* gene product CpsA acts as a transcriptional regulator of *cps* loci by specifically binding to the *cps* promoter sequences and this has been shown for both *Streptococcus iniae* and *Streptococcus agalactiae* (Hanson et al., 2010; Hanson et al., 2012). However, this conclusion has not been confirmed in the D39 strain. Additional studies have shown that CpsA is linked to CPS attachment to cell wall peptidoglycans (Stefanović et al., 2021). The expression of *cps* loci is regulated via specific transcription factor expression and responses to environmental signals (Xiao et al., 2021). Transcriptional regulation is essential for *S. pneumoniae* to adapt to changes in the host environment (Aprianto et al., 2018) and involves two-component signal transduction systems (Standish et al., 2005; Yu et al., 2023). A previous study had determined that mRNA levels from the *cps* locus in strain D39 were upregulated in the bloodstream during pneumococcal infection (Ogunniyi et al., 2002). Together, these data suggest that regulation of *cps* transcription can be influenced by environmental conditions.

In previous studies, we identified that the protein SPD_0410 can bind to the *cps* promoter in DNA pull-down experiments and this suggested its potential involvement in CPS synthesis (Wu et al., 2016). In the current study, we focused on whether the SPD_0410 protein is a regulator of CPS. We observed that the absence of *spd0410* led to increased CPS levels on the bacterial surface and inside the cells implicating this protein as a negative regulator. EMSA and DNase I footprinting analyses confirmed that SPD_0410 specifically binds to two distinct sites within the *cps* promoter region and therefore functions as a novel factor that can regulate the *cps* gene locus. However, surprisingly, our transcriptome sequencing did not reveal significant changes for the *cps* gene locus and this may be attributed to samples taken from the logarithmic growth phase and agrees with the qRT-PCR findings. Since we found significant upregulation of mRNA levels from the *cps* locus in the early stages of growth in D39Δ*spd0410*, we suspect that SPD_0410 may be responding to new environmental or nutritional changes in the early stages of growth. Thus, these factors would trigger specific regulatory mechanisms of the *cps* gene clusters. However, this regulation may be adjusted or alleviated as bacteria progress into the logarithmic growth phase. Nevertheless, we consider this data to be significant as it highlights the complexity of bacterial physiology.

Previous studies have indicated that a group of *cps* transcriptional activators typically possess conserved domains that enable them to function as transcription factors, particularly as global transcriptional regulators (Moscoso and García, 2009; Solano-Collado et al., 2016). However, our comparison of the amino acid sequence of SPD_0410 revealed that its orthologs were hypothetical proteins with no detailed reports on their biological functions. Surprisingly, we found that SPD_0410 in *S. pneumoniae* exhibits a certain similarity to its homologous protein containing a helix-turn-helix structure. This suggests that SPD_0410 may also bind to specific DNA sites in a dimeric form. This hypothesis is supported by our gel shift and footprinting experiments that demonstrated SPD_0410 binding to the *cps* promoter at two specific sites. These findings provide insights for the further investigation of the complete biological function of SPD_0410.

The growth of *S. pneumoniae* is governed by carbon catabolite repression via the transcriptional regulator CcpA. As such, the carbon source influences capsule synthesis and thickness across different serotypes (Iyer et al., 2005; Görke and Stülke, 2008). This links carbohydrate metabolism and optimization of conditions necessary for survival within the host and enhanced virulence (Iyer et al., 2005). This regulatory mechanism illustrates how bacteria adjust their infection capabilities through environmental adaptation and metabolic adjustments. Glucose may serve as a host-derived signal that activates the expression of virulence genes in the pneumococcus, allowing it to adapt to different microecological environments (Moscoso and García, 2009; Troxler et al., 2019). In the serotype 4 strain TIGR4, the TIGR4Δ*ccpA* mutant significantly reduced nasopharyngeal colonization and pulmonary infection. This diminished virulence may be attributable to the role of extracellular glucose concentration in positively modulating CPS biosynthesis levels (Giammarinaro and Paton, 2002; Iyer et al., 2005). We noted significant changes in metabolic pathways in our transcriptome sequencing results, including glycolysis and gluconeogenesis.

Glucose is the preferred carbohydrate for *S. pneumoniae* so we speculate that the regulation of CPS by SPD_0410 is linked to glucose metabolism. Interestingly, the growth and morphology of the D39s and D39Δ*spd0410* strains at differing glucose concentrations were similar. Previous studies have shown that damage to key cell-separating enzymes such as LytA, leads to incomplete cleavage of peptidases between daughter cells, resulting in the formation of long chains (Dalia and Weiser, 2011). Additionally, the absence of ChoP can inhibit the dissociation of daughter cells during cell division (Zeng et al., 2014). Our transcriptome data did not reveal changes in these related genes which may explain why the strains did not show significant differences in growth and morphology. CPS expression and activity of the *cps* promoter varied in response to glucose levels for the D39s but not D39Δ*spd0410*. We also observed similar results in the *cps* promoter mutant strain. These results link SPD_0410 to glucose and the regulation of CPS expression. However, we found no significant differences in *spd0410* mRNA levels at differing glucose levels (Supplementary Figure 5). Whether SPD_0410 functions similarly to known glucose-sensing regulators (Wu et al., 2016; Suo et al., 2023) remains uncertain and warrants further investigation in future studies.

The capsule is crucial for the resistance of *S. pneumoniae* to host immune system invasion and macrophage phagocytosis. Our experimental results indicated that D39Δ*spd0410* possessed enhanced resistance to macrophage phagocytosis compared to the parental strain, attributable to elevated capsule synthesis. In addition, D39Δ*spd0410* was less adherent to cell surfaces, consistent with prior studies indicating that a thickened capsule can obscure bacterial surface adhesion factors, thereby diminishing bacterial adhesion (Hammerschmidt et al., 2005; Shak et al., 2013).

In the mouse intranasal infection assay, the D39Δ*spd0410* colonized to a lesser extent than D39s in the nasopharynx but was more likely to invade organs and blood, causing systemic infection. Although the number of CFU recovered from tissues was statistically higher for D39Δ*spd0410* than for the parental strain D39s, the actual differences were minor. This indicates that while the deletion of the *spd0410* gene alters colonization dynamics, the magnitude of the change in CFU numbers is relatively modest. Capsular synthesis undergoes dynamic changes during bacterial colonization, potentially linked to phase transitions between different capsular phenotypes (opaque and transparent) (Saluja and Weiser, 1995; Shainheit et al., 2014). The nasopharyngeal environment can harbor environmental signaling molecules that decrease CPS expression while increasing adhesion factor expression (Kim and Weiser, 1998; Weiser, 1998). However, following the initial colonization phase, bacteria upregulate capsule expression to facilitate more effective lung invasion. In this process, the capsule diminishes complement deposition on the bacterial surface and significantly enhances anti-phagocytic resistance (Kadioglu et al., 2008; Hyams et al., 2010). Notably, the survival curve demonstrates clear biological differences resulting from the deletion of the *spd0410* gene, underscoring that, despite modest CFU differences, the mutant strain has a distinct pathogenic profile characterized by increased systemic invasion potential.

In summary, this study revealed for the first time the mechanism of SPD_0410 as a novel regulator of *S. pneumoniae* capsule synthesis. SPD_0410 functions as a negative regulator of the *cps* gene locus and modulates virulence and host affinity by regulating CPS synthesis. Furthermore, the association of SPD_0410 with glucose metabolism suggests this may be a fruitful direction for further research to unravel the functions of SPD_0410.

## Data Availability

The data presented in the study are openly available in figshare at https://figshare.com/s/c755a69f86e12c110f7c.
