# Supplementary material for: SPD_0410 negatively regulates capsule polysaccharide synthesis and virulence in Streptococcus pneumoniae D39
Source: Front Microbiol. 2025 Jan 3;15:1513884. doi: 10.3389/fmicb.2024.1513884 (PMC11739294; doi:10.3389/fmicb.2024.1513884)
Supplement: Supplementary file 1 [file Data_Sheet_1.docx]

**Supplemental Material**


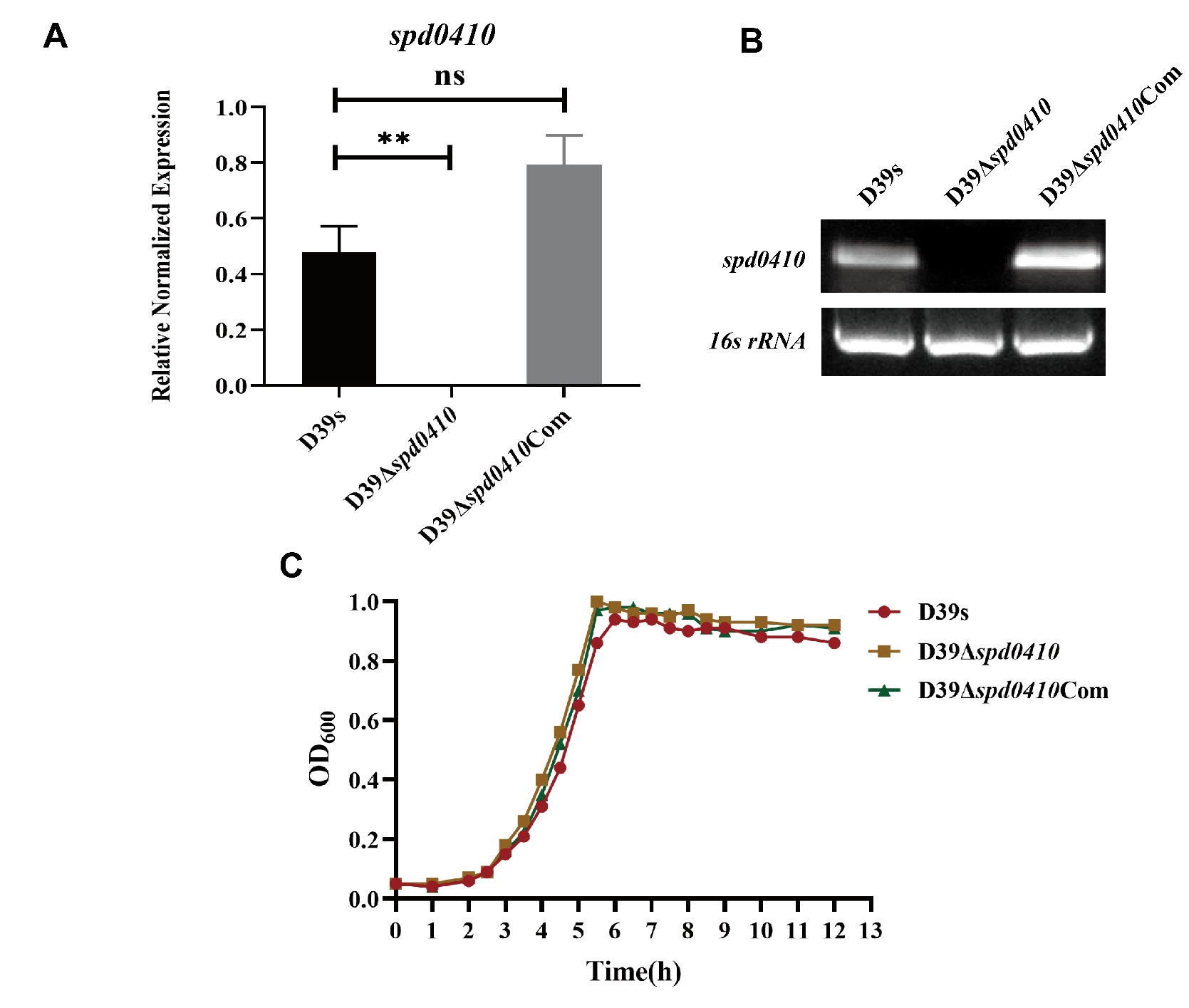


**Figure S1.** **Construction and growth observation of mutant strains.**

(A)The *spd0410* mRNA of strains D39s, D39Δ*spd0410*, and D39Δ*spd0410*Com were determined by real-time quantitative PCR (qPCR). Relative mRNA levels were expressed relative to that of *gyrB*. The results of representative experiments were presented as the mean of three replicates ± SD, each determined in triplicate. **P < 0.01; ns, not significant, as analyzed by unpaired two-tailed Student’s t-test. (B) Verification of the D39Δ*spd0410* and D39D39Δ*spd0410*Com strains using the nucleic acid electrophoresis gel. *16S rRNA* was used as a reference. (C) Observation of growth curve of related strains in C+Y medium.


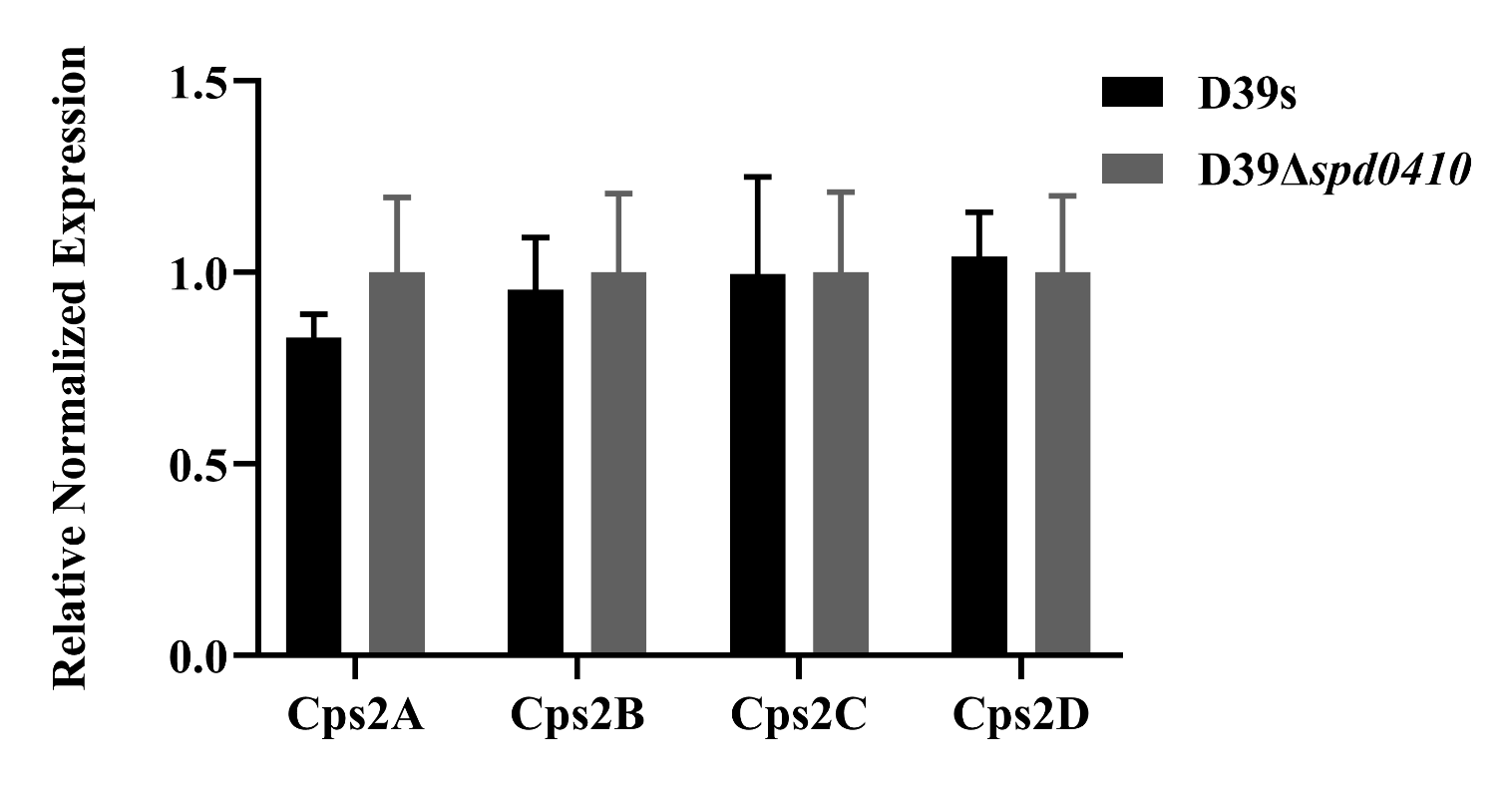


**Figure S2.** Relative mRNA levels of *cps*2A-D in early stages of bacterial logarithmic growth (OD_600nm_=0.5), the first four genes downstream of the *cps* operon in the strains. Relative mRNA levels were expressed relative to that of *gyrB*. The results of representative experiments were presented as the mean of three replicates ± SD, each determined in triplicate.


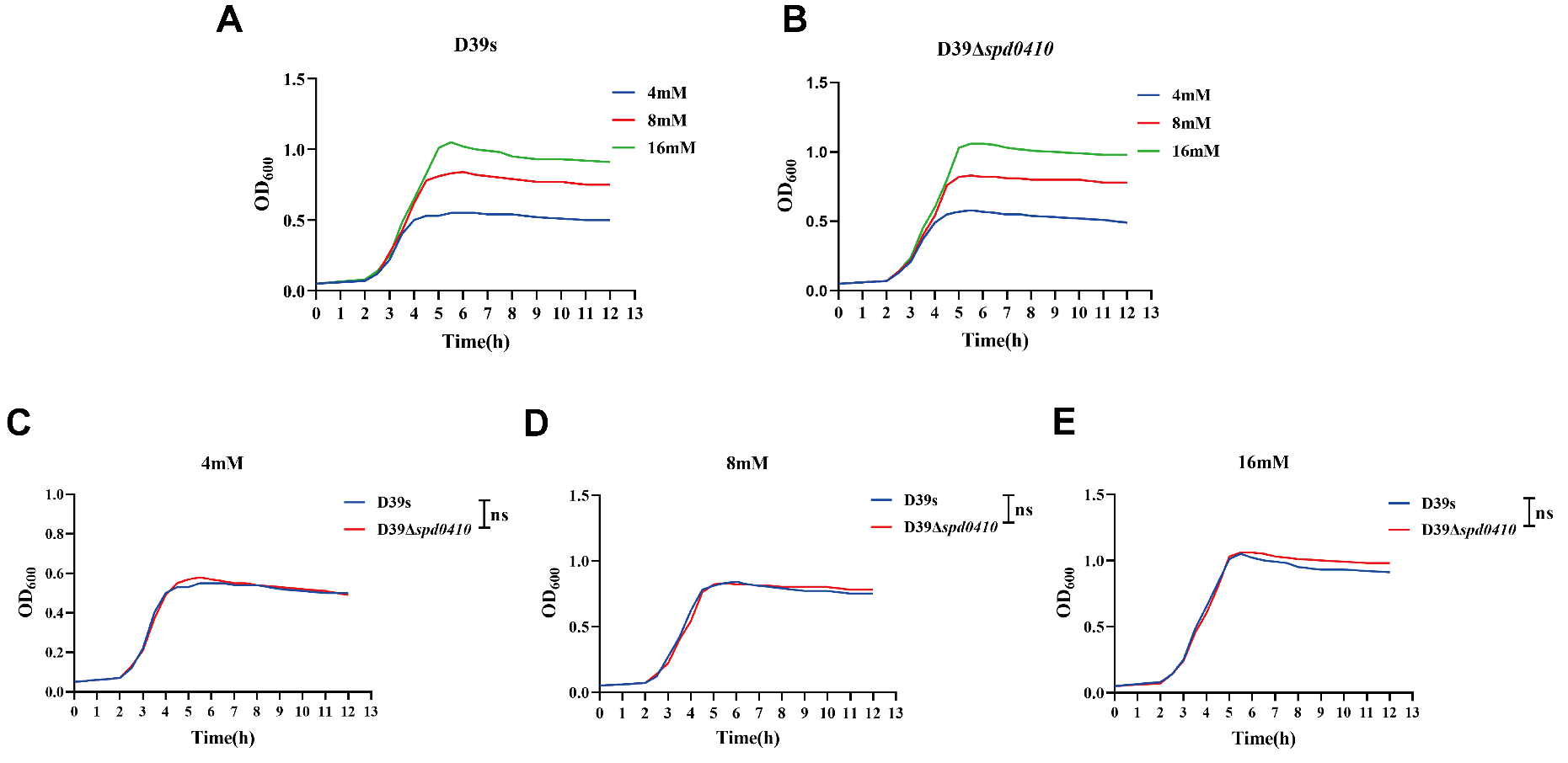


**Figure S3.** **Growth curves of strains in C+Y medium with different glucose concentrations.**

（A）Growth curves of parent strain D39s at different glucose concentrations. (B) Growth curves of defective strain D39Δ*spd0410* at different glucose concentrations. (C to E) Comparison of growth curves of D39s and D39Δ*spd0410* at different glucose concentrations: (C) 4mM, (D) 8mM,（E）16mM. NS, not significant, as analyzed by unpaired two-tailed Student’s t-test.


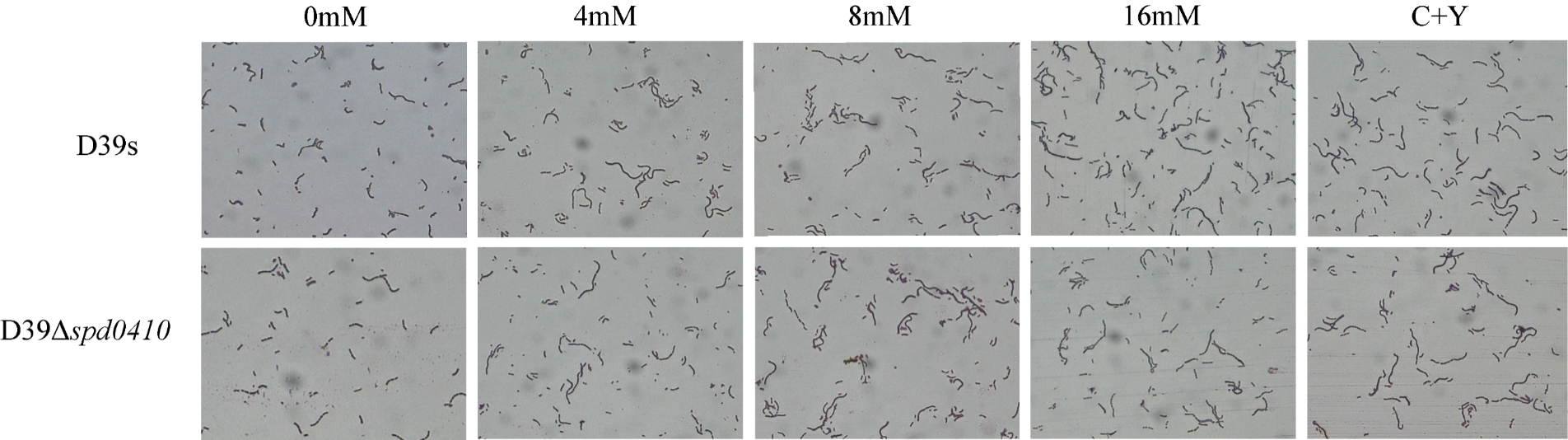


**Figure S4.** Gram staining was used to observe the bacterial morphology at different glucose concentrations (×40 objective).


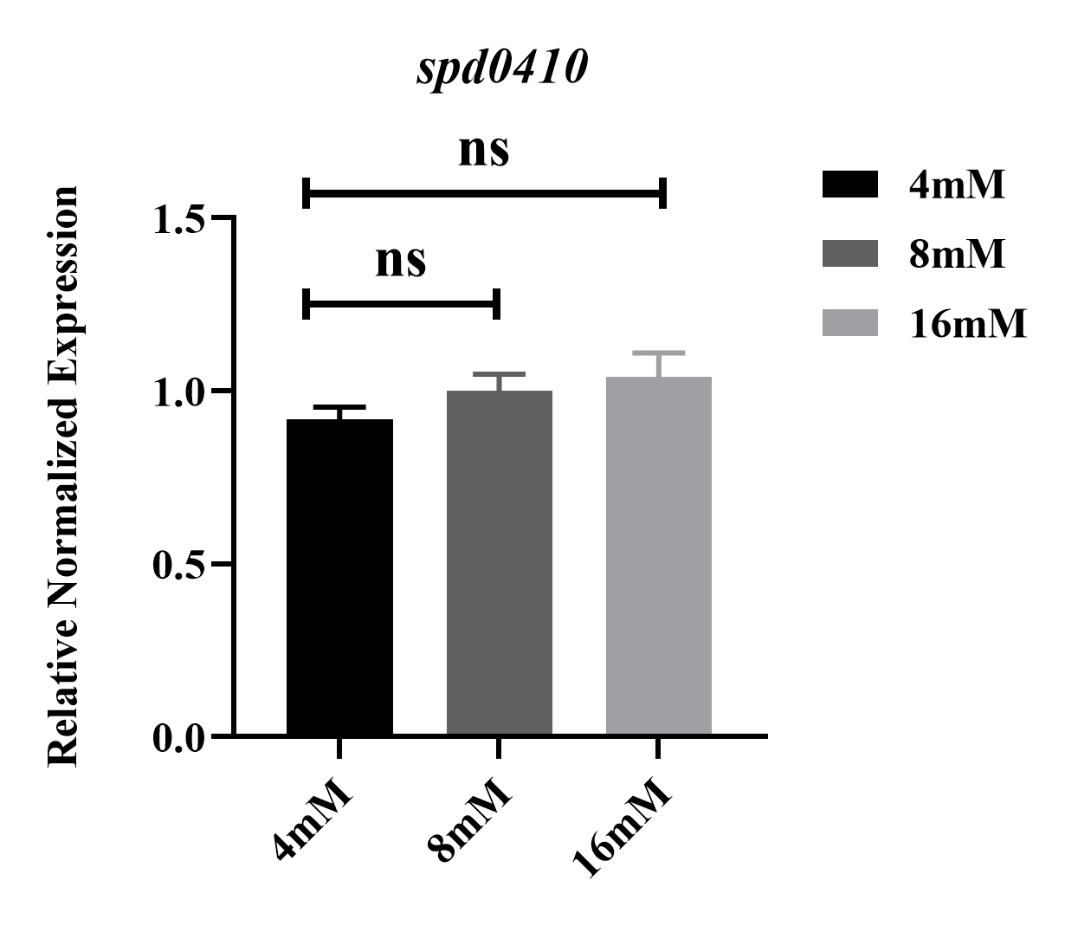


**Figure S5.** Relative mRNA levels of the *spd0410* mRNA expression in strain D39s under different glucose concentrations. Relative mRNA levels were expressed relative to that of *gyrB*. The results of representative experiments were presented as the mean of three replicates ± SD, each determined in triplicate. NS, not significant, as analyzed by unpaired two-tailed Student’s t-test.

**Table S1.** **Primers used in this study**

| **Primer** |  | | **Primer Sequence (5´-3´)** |
| --- | --- | --- | --- |
| JC  Erm F  Erm R  D39Δspd0410up  D39Δspd0410down  D39Δspd0410Com  ΔPcps::Jc  Pcps-Mut  Pcps-Mut 1  Pcps-Mut 2  Pcps F  Pcps R  gyrB    spd0410  Cps2A    Cps2B  Cps2C  Cps2D | Pr1332  Pr1333  P1  P2  P3  P4  Pr9626  Pr9627  Pr9020  Pr9026  Pr9027  Pr9023  Pr9028  Pr9034  Pr9033  Pr9029  Pr9028  Pr9021  Pr9022  Pr9029  Pr9028  Pr9025  Pr9024  Pr9029  Pr9030-bio  Pr9031  Pr9032  gyrB F  gyrB R  spd0410 F  spd0410 R  Cps2A F  Cps2A R  Cps2B F  Cps2B R  Cps2C F  Cps2C R  Cps2D F  Cps2D R | TCTAGAGGATAATGCTGAAAACTCCTTGAAG  CTCGAGCCTTTCCTTATGCTTTTGGAC  CCGGGCCCAAAATTTGTTTGAT  AGTCGGCAGCGACTCATAGAAT  Ccaaaggagtgatgaatttgaagg  atcaaacaaattttgggcccggGCCATGTTTTTCTCCTATTTTC  attctatgagtcgctgccgactTCTTTGGATAACGGCGGAT  Gaaaacctggaaaaggtcggcat  Ggaagatcttccatggctaagtcaaactttg  CCGCTCGAGCGGttaatcttcaaatttttcataatt  cgcttcctagttgtggctaact tttcagcattatcctctagaggcaaagtatctggaagaag gcataaggaaaggctcgagttacgcaactgacgagtgtg tcagtgcttcggttgacgttaat  ccaaggtgaggagattgggatg  cccgcgaaccaaaaatatagtaaaatgaaataagaacatgac tgactgtcctgatcaatttgtcatgttcttatttcattttac  ctcttgacttgggaccgtcatc  ccaaggtgaggagattgggatg TTCTAATGCTATTTTAACGTCAGAtgtgtactattctagtttc  TCTGACGTTAAAATAGCATTAGAAaacgatttgactgtcctg Ctcttgacttgggaccgtcatc  ccaaggtgaggagattgggatg CACTATAGATATCTTTAGTCGTCTTagtagacttcccgcgaac  AAGACGACTAAAGATATCTATAGTGaaaaaaggtgtagacattac  ctcttgacttgggaccgtcatc  tacacaTCTGACGTTAAAATAGC  tacacaTCTGACGTTAAAATAGC  Acgtgtgatgcttctgttat  GTTCGTATGCGTCCAGGGAT  ATACCACGCCCATCATCCAC  GTAGAATCAGTTGTTGGCTGGG  GCGCCAGAGCAATGATAGAC  TTGTCAGCTCTGTGTCGCTC  TTATCAGTCCCAGTCGGTGC  CTACCTCTCACCGTCGCAAG  CAGCCCCGTAAGCAATGACT  AAACAGCCAGAGGAAGCCAG  GAAGGAGTCGTAGCTGGTCG  TCCTGTCGGTGTCGTGATTG  AAACGGCTTCCCTGTGTGTT | |
